# A decision support model for investment on P2P lending platform

**DOI:** 10.1371/journal.pone.0184242

**Published:** 2017-09-06

**Authors:** Xiangxiang Zeng, Li Liu, Stephen Leung, Jiangze Du, Xun Wang, Tao Li

**Affiliations:** 1 School of Information Science and Technology, Xiamen University, Xiamen, China; 2 Faculty of Engineering, University of Hong Kong, Hong Kong, China; 3 School of Finance, Jiangxi University of Finance and Economics, Nanchang, China; 4 College of Computer and Communication of Engineering, China University of Petroleum, Qingdao, China; 5 School of Computer Science, Florida International University, Miami, FL, United States of America; Tianjin University, CHINA

## Abstract

Peer-to-peer (P2P) lending, as a novel economic lending model, has triggered new challenges on making effective investment decisions. In a P2P lending platform, one lender can invest *N* loans and a loan may be accepted by *M* investors, thus forming a bipartite graph. Basing on the bipartite graph model, we built an iteration computation model to evaluate the unknown loans. To validate the proposed model, we perform extensive experiments on real-world data from the largest American P2P lending marketplace—Prosper. By comparing our experimental results with those obtained by Bayes and Logistic Regression, we show that our computation model can help borrowers select good loans and help lenders make good investment decisions. Experimental results also show that the Logistic classification model is a good complement to our iterative computation model, which motivates us to integrate the two classification models. The experimental results of the hybrid classification model demonstrate that the logistic classification model and our iteration computation model are complementary to each other. We conclude that the hybrid model (i.e., the integration of iterative computation model and Logistic classification model) is more efficient and stable than the individual model alone.

## Introduction

Peer-to-peer (P2P) lending is an emerging financial market. In recent years, more people are engaged in this financial platform. For example, the volume of business and the turnover of Prosper and Lending Club, the large-scale online P2P lending intermediary agents in the United States, are 50 million and nearly 100 million per month, respectively. The P2P lending is developing rapidly all over the world. Examples of P2P lending include PPDai in China and Zopa in Europe. In Prosper, individuals either request to borrow money, take a borrower role, or buy loans as a lender. The borrower sets the amount of money he or she needs and the maximum rate he or she would be willing to pay for this loan by posting a listing. Each lender will scan these loans to bid a partial amount and give the minimum rate they are willing to receive. The main difference between P2P lending and traditional bank industry is that in the former, each lender can not only obtain the loan's financial information, but also evaluate the risk of bidding according to the borrower's social characteristic. On the other hand, Prosper, as an intermediary agent of P2P lending, collects many borrowers and lenders, help users borrow money quickly or gain benefits by investing. In this Internet platform, investors and borrowers form an *M*-to-*N* relation model called bipartite graph, in which a lender can invest *N* loans, and a loan may be accepted by *M* investors.

P2P lending, as a burgeoning financial market, becomes a new field for academic research. In recent years, the social networking services on P2P lending have been explored extensively. Berger and Gleisner [1] found that these market participants act as financial intermediary and can significantly improve borrowers' credit conditions by reducing information asymmetries, predominantly for borrowers with less attractive risk characteristics. Freedman and Jin [2] examined what information problems exist on Prosper and whether social networks can help alleviate the information problems. They found that the estimated returns of groups loans are significantly lower than those of non-group loans partially due to lender learning and partially due to Prosper eliminating group leader rewards. Lin et al. [3] tested whether social networks lead to better lending outcomes, focusing on the distinction between the structural and relational network measures that are associated with a higher likelihood of a loan being funded, a lower risk of default, and lower interest rates. The social networks approaches were also applied in other fields, such as bioinformatics [4]. Collier and Hampshire [5] draw on the theory from the Principle-Agent perspective to empirically examine the signals that enhance community reputation. Sergio [6] measured the influence of social interactions in the risk evaluation of a money request; with a special focus on the impact of one-to-one and one-to-many relationships. His results showed that fostering social features increases the chances of getting a loan fully funded, when financial features are not sufficient to construct a differentiating successful credit request. Chen et al. [7] described an approach to measure the entrepreneurship orientation of online P2P lending platforms.

Some researchers take the perspective of borrowers when developing a model. Wu and Xu [8] proposed a decision support system based on intelligent agents in P2P lending for borrowers. The system provides borrowers with individual risk assessment, eligible lender search, lending combination and loan recommendation. The empirical results in [9] displayed that borrower' decisions, such as loan amount and interest rate, will determine whether he or she could successfully find loans or not. Herzenstein et al. [10] gave specific suggestions to borrowers to increase their chances of receiving funding in P2P lending communities. Pope and Sydnor [11] discovered that loan listings with blacks in the attached picture are 25% to 35% less likely to receive funding than those of whites with similar credit profiles.

To help investors make better investment decision, Luo et al. [12] proposed a data driven investment decision-making framework, which exploits the investor composition of each investment for enhancing decisions in P2P Lending. Katherine and Herrero-Lopez [13] examined the behavior of lender in a large peer-to-peer lending network and find that, while there exists high variance in risk-taking between individuals, many transactions represent sub-optimal decisions on the part of lenders. Lauri et al. [14] introduced a Borrower Decision Aid which helps formalize the decision making process of the sellers, or borrowers. Singh et al. [15] focused on risk and return of investments on Prosper. They found that within each credit grade, there exist subgroups which give positive return. For these subgroups, risk is aligned with return. In addition, the groups of loans with lower credit grades are more efficient in terms of risk and return alignment than those with higher credit grades. Klafft [16] demonstrated that following some simple investment rules may improve profitability of a portfolio and lead to acceptable returns for all credit rating categories with an exception of the high-risk ones. Garman et al. [17] introduced the concept of a research premium, which is the difference between the interest rate borrowers would pay in a market with costless search and the minimum interest rate they must offer to induce search. Iyer et al. [18] evaluated whether lenders in such peer-to-peer markets are able to use borrower information to infer creditworthiness. They examined this capability using a methodology that takes advantage of the category of the borrowers and found that lenders are able to use available information to infer creditworthiness that is captured by a borrower's credit score.

P2P lending involves diverse elements, which renders research opportunities and challenges that are under-explored currently. The goal of this paper is to predict new investment abilities of investors and filter good requests in new loans on the basis of the bipartite graph model. This work focuses on synthesizing those old manifestations of investors in the past investments and providing a comprehensive evaluation. The performance of old investors' will help discover and analyze good new loans and reliable new investments according to the lending and investing model generated from P2P lending data.

The organization of this paper is as follows: Section 2 defines a *M*-to-*N* relation model called bipartite graph, which is based on the relation of investors and loans in P2P lending. Section 3 quantitatively analyzes the comprehensive evaluation of new investors and unknown status loans by modeling an iteration computation model. Section 4 gives an integrated decision model to help investors pick trustworthy loans. Section 5 validates the effectiveness of the proposed investment decision model on the basis of real world data from the Prosper platform. By comparing current experimental results with those obtained by BayesNet [19], Logistic [20], and Average, we show that our computation model can help investors make better investment decisions. In addition, our empirical results demonstrate that the logistic classification model and the proposed iteration computation model complement each other. As a result, the hybrid model (i.e., the integration of our model and Logistic model) is more efficient and stable than each of the individual model. Finally, Section 6 presents conclusions of this work.

## Comprehensive evaluation of investor and load

### Investing network

P2P lending on Prosper is similar to that in the stock market, in which each investor can disperse his or her money to numerous loans and relatively one loan may be allocated by a multi-lender. This many-to-many relationship can be modeled as a *bipartite graph* which is widely used to model the relationship between two types of entities. We will use the *bipartite graph* to explain the relationship of investors and investees.

In the bipartite graph of P2P lending relationship, investors and borrowers are different types of entities, and the weight of edges is the amount of investors bidding for loan. In particular, on the Prosper online platform, if one borrower needs to borrow a sum of money, he or she must apply for a loan. After the application is approved by Prosper, a listing will be posted for all investors. Each lender who scans the loan can bid a partial amount until completed in full.

**Fig 1:**
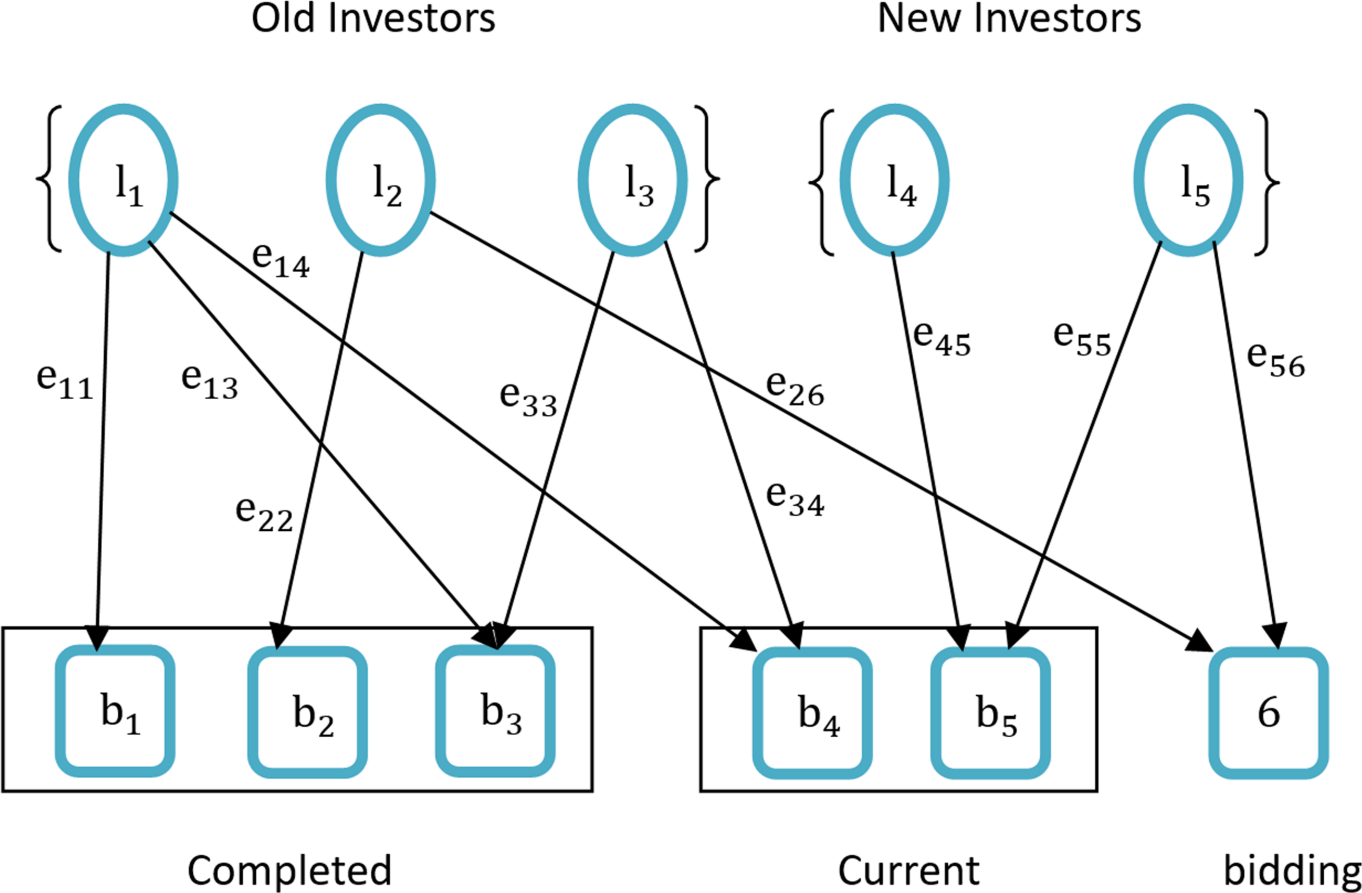
Simple bipartite graph of investment network.

We use *L* = {*l*_1_,*l*_2_,⋯,*l*_*n*_} to represent the set of *N* investors, and *B* = {*b*_1_,*b*_2_,⋯,*b*_*m*_} stands for *M* borrowers. *S* = {*status*_1_,*status*_2_,⋯,*status*_*m*_} represents the status of *M* loans and *status*_*m*_ = {1,0}. *e*_*ij*_ is the amount that investor *l*_*i*_ has lent to borrower *b*_*j*_ and is equal to *0* if no correlation exists. Thus, *E* = {*e*_11_,*e*_12_,⋯*e*_*ij*_,⋯*e*_*nm*_} is the set of all biddings. Finally, *L*, *B*, and *E* make up a bipartite investment network *G* = {*L*,*B*,*E*}. Fig 1 shows an example of this relation.

### Initial evaluation model

Basing on the bipartite graph of the investment network, we can propose a comprehensive evaluation model for investors and borrowers.

First, to judge whether a lender is a reliable investor, we need to determine his or her investment performance, which is equal to the investment amount and income that serve as moderator attributes. We use *LS*_*i*_ to stand for the confidence level of lender *l*_*i*_. Note that *status*_*j*_ = {0,1} shows the status of loan *b*_*j*_. Therefore, *LS*_*i*_ is the ratio of investor *l*_*i*_'s total amount of paid investment loans to the total amount of investment loans in the investment network. So *LS*_*i*_ can be written as Eq (1):

$${LS}_{i}=\frac{\sum_{j=1}^{m}e_{ij}*{status}_{j}}{\sum_{j=1}^{m}e_{ij}},i=1,2,\cdots n$$ (1)

where *m* is the number of completed loans and *n* is the number of old investors.

When investors who have finished some biddings are evaluated, we can rely on their confidence level to analyze the current and bidding loans to which they have allocated money. We use *BS*_*j*_ to stand for the predicted paid probability of the current or bidding loan *b*_*j*_, which can be computed by Eq (2):

$${BS}_{j}=\frac{\sum_{i=1}^{n}e_{ij}*{LS}_{i}}{\sum_{i=1}^{n}e_{ij}},j=1,2,\cdots m$$ (2)

where *n* represents the quantity of investors and m is the quantity of current and bidding loans.

**Fig 2:**
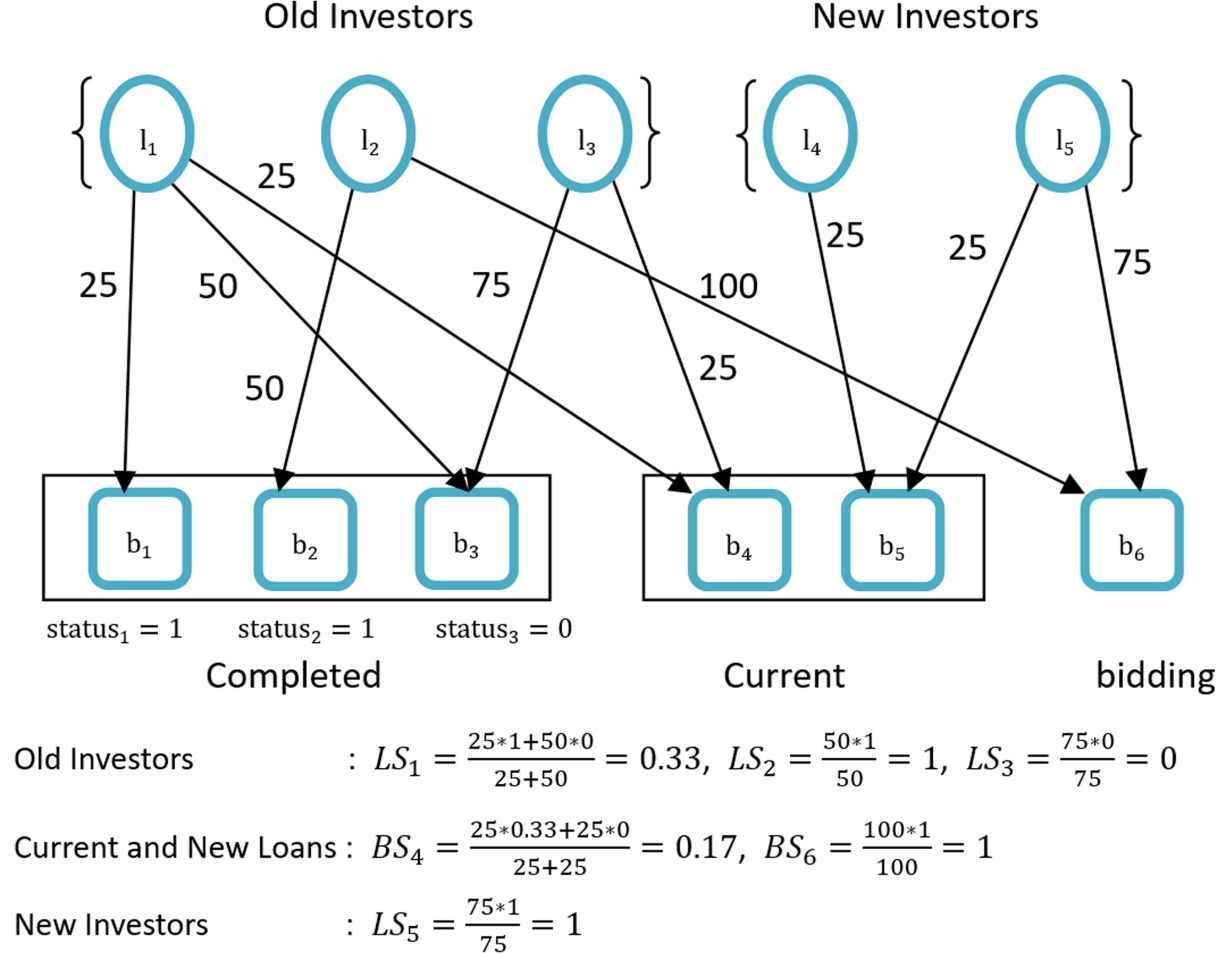
Initial evaluation.

Basing on the paid probability of the current or bidding loans, we will evaluate the confidence level of novices, as written in Eq (3):

$$new\;{LS}_{i}=\frac{\sum_{j=1}^{n}e_{ij}*{BS}_{j}}{\sum_{j=1}^{n}e_{ij}},i=1,2,\cdots m$$ (3)

where *n* is the number of current and bidding loans, and *m* is the number of new investors. A simple case of initial evaluation is given in Fig 2.

A problem exists with this initial evaluation. In the above case, we can observe that the confidence level *LS*_4_ of investor *l*_4_ and the paid probability *BS*_5_ of loan *b*_5_ are not evaluated. However, by carefully analyzing the result and the bipartite graph of the investment network, we learnt that *BS*_5_ can be evaluated by the relationship with investor *l*_5_. Similarly, now that *BS*_5_ is known, *LS*_4_ can also be calculated according to the relationship with loan *b*_5_. Therefore, we need to conduct a second round of calculation base on initial computation results.

### Second round evaluation

**Fig 3:**
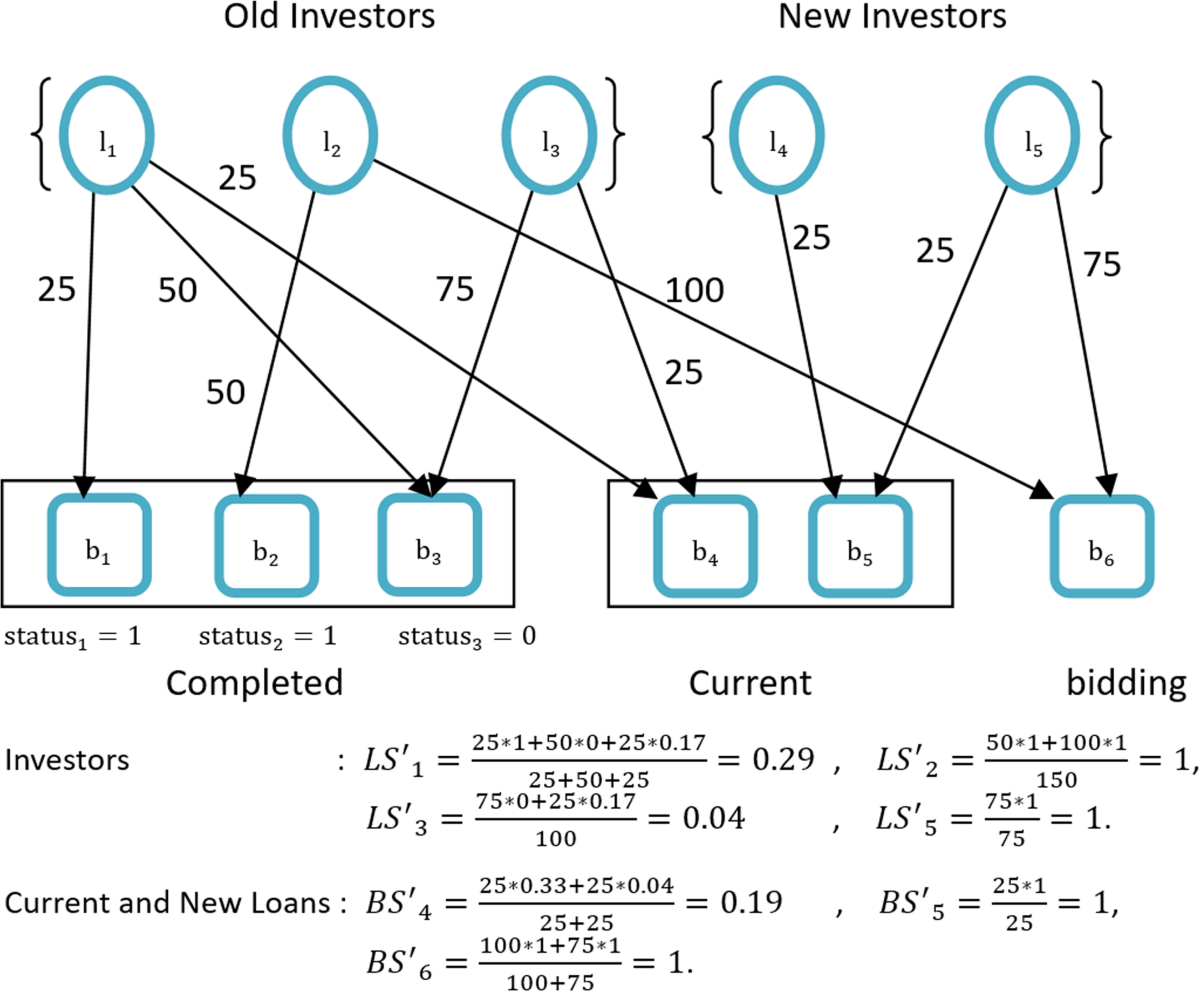
Second evaluation.

Some current and bidding loans evaluated from the first round of calculation are evaluated in the second round of evaluation. Therefore, to evaluate the old investor's *LS*′_*i*_ again, we should employ the paid, current, and bidding loans:

$${{LS}^{\prime }}_{i}=\frac{\sum_{j=1}^{m}e_{ij}*{status}_{j}+\sum_{j=m+1}^{M}e_{ij}*{BS}_{j}}{\sum_{j=1}^{M}e_{ij}},i=1,2,\cdots n$$ (4)

where *m* is the number of completed loans, *M* is the number of all loans and *n* is the number of all investors. Therefore, Eq (3) is not needed in the calculation, and the current and bidding loans' paid probability *BS*′_*j*_ in the second round can be written as:

$${{BS}^{\prime }}_{j}=\frac{\sum_{i=1}^{n}e_{ij}*{{LS}^{\prime }}_{i}}{\sum_{i=1}^{n}e_{ij}},j=1,2,\cdots m$$ (5)

where *n* represents the quantity of investors and *m* is the quantity of current and bidding loans. Therefore, by using Eq (5), *BS*′_5_ = 25 * 1/25 = 1. The integrated results are shown in Fig 3.

### Multi-round and convergence

**Fig 4:**
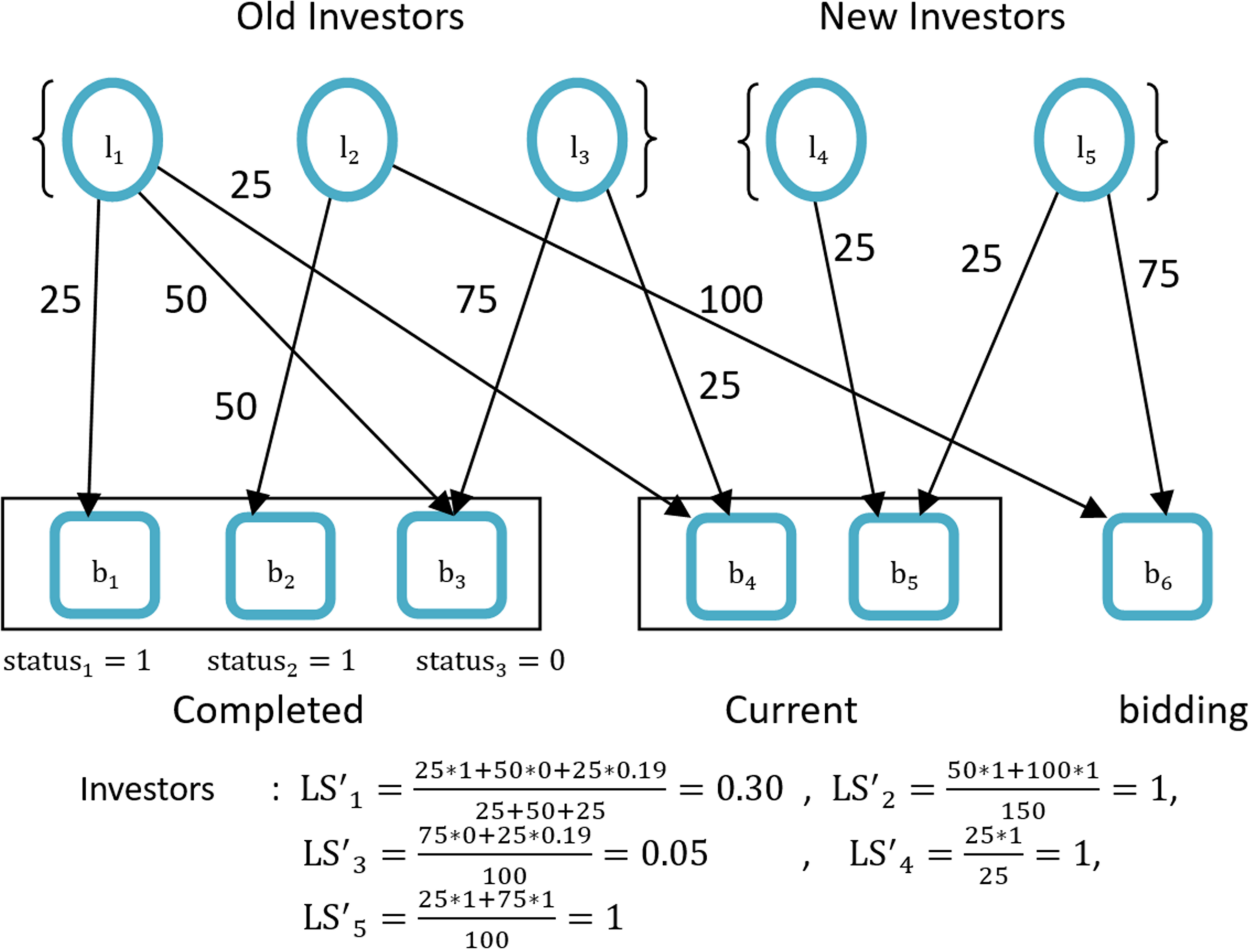
Investor confidence level in the third round.

**Fig 5:**
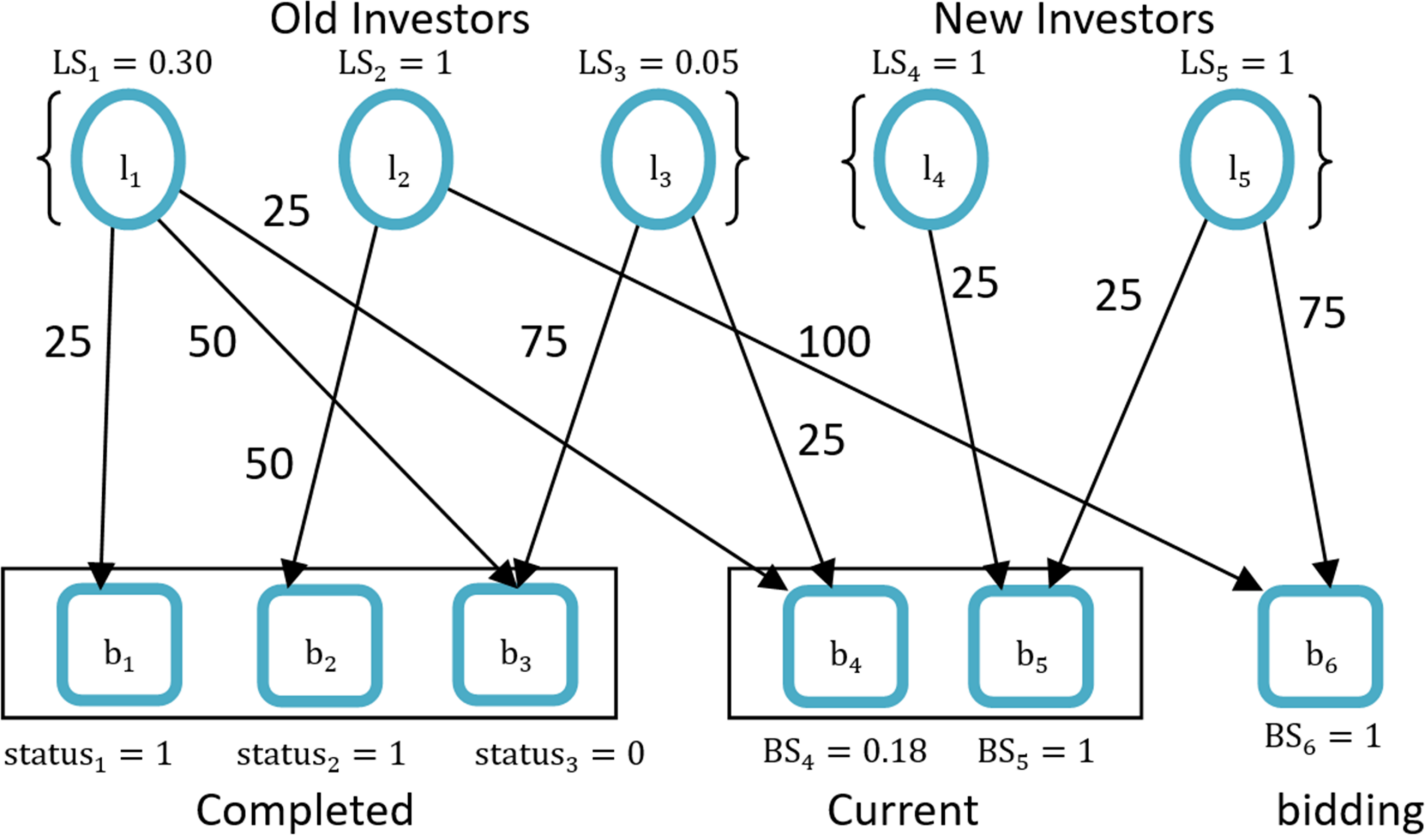
Convergence state of a simple case.

Unfortunately, investor *l*_4_ is still not correlated and evaluated. Nevertheless, this problem is not serious, because we can easily predict the value of *l*_4_ by executing Eq (4) again. We obtain *LS*′_4_ = 25 * 1/25 = 1. Fig 4 shows the results of the investors' confidence level in the third round. Eqs (4) and (5) are recursive. Therefore they can be computed with initial states and by iterating the computation until convergence. Fig 5 demonstrates a consistent steady state solution for an investment network.

### Convergence properties

**Fig 6:**
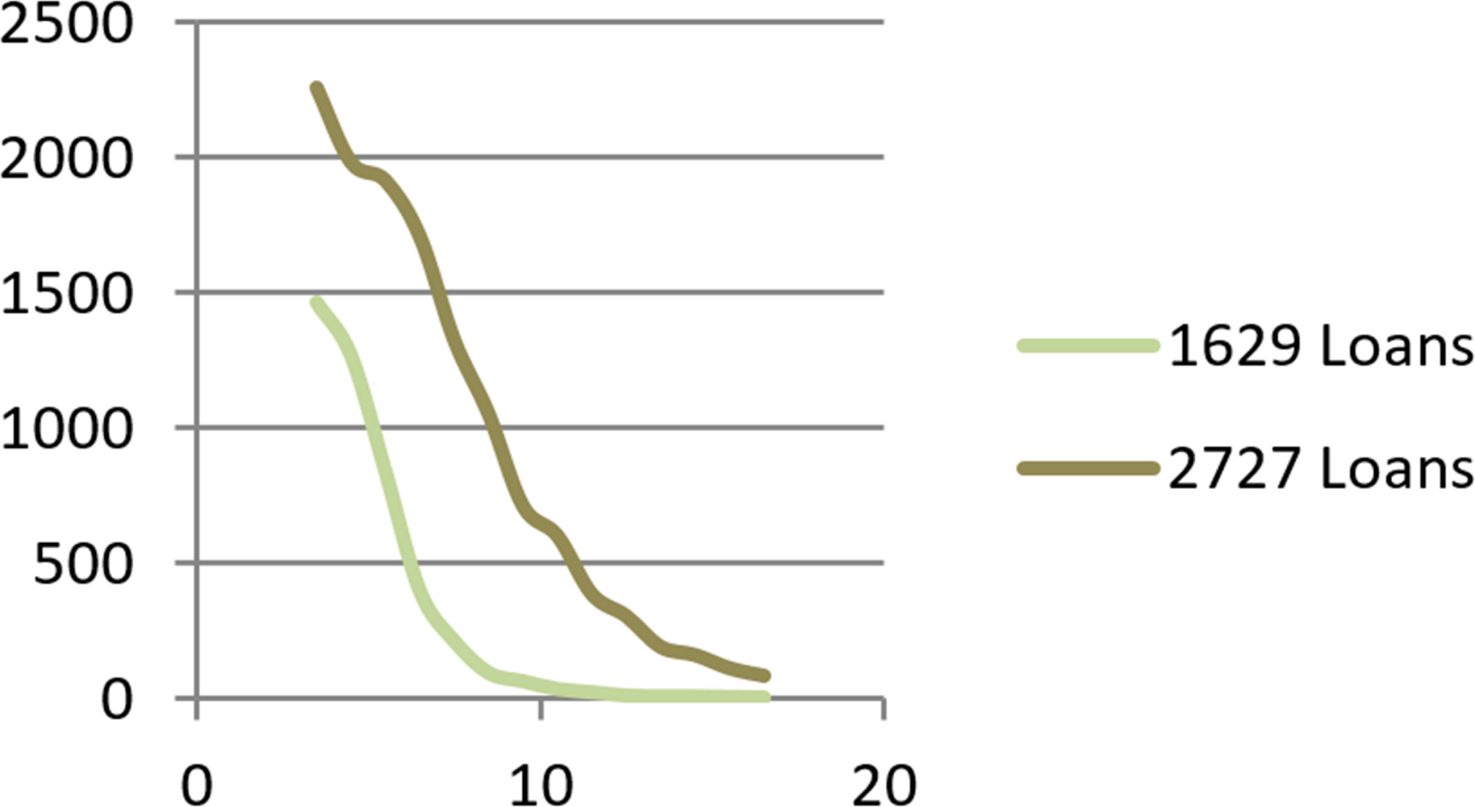
Rate of convergence with different numbers of loans.

In Fig 5, all investors and loans converge to a steady state. In this work, we use real data from Prosper that includes more than 300,000 bids, more than 4,000 loans, and more than 12,000 investors. The evaluation process takes 25 iterations to converge. Fig 6 shows the rate of convergence with different numbers of loans. The graph suggests that our computation model scale well even for large collections as the scaling factor is roughly linear in *log n*. This computation process is similar to the PageRank [21] computation with the convergence property as described in [22]. Our model computation terminates in logarithmic time, which is equivalent to saying that the bipartite graph has a good expansion factor.

## Decision model

In the P2P lending market on Prosper, there are many loans that are posted at same time; and we need to assess those loans whether they will be paid. In the stock market, smart investors usually make good selections (i.e., wise decisions). Similarly, in the P2P lending market, investors have different knowledge backgrounds and may make good or bad decisions. Therefore, good investors are our reference objects before making a decision.

### Model description

**Fig 7:**
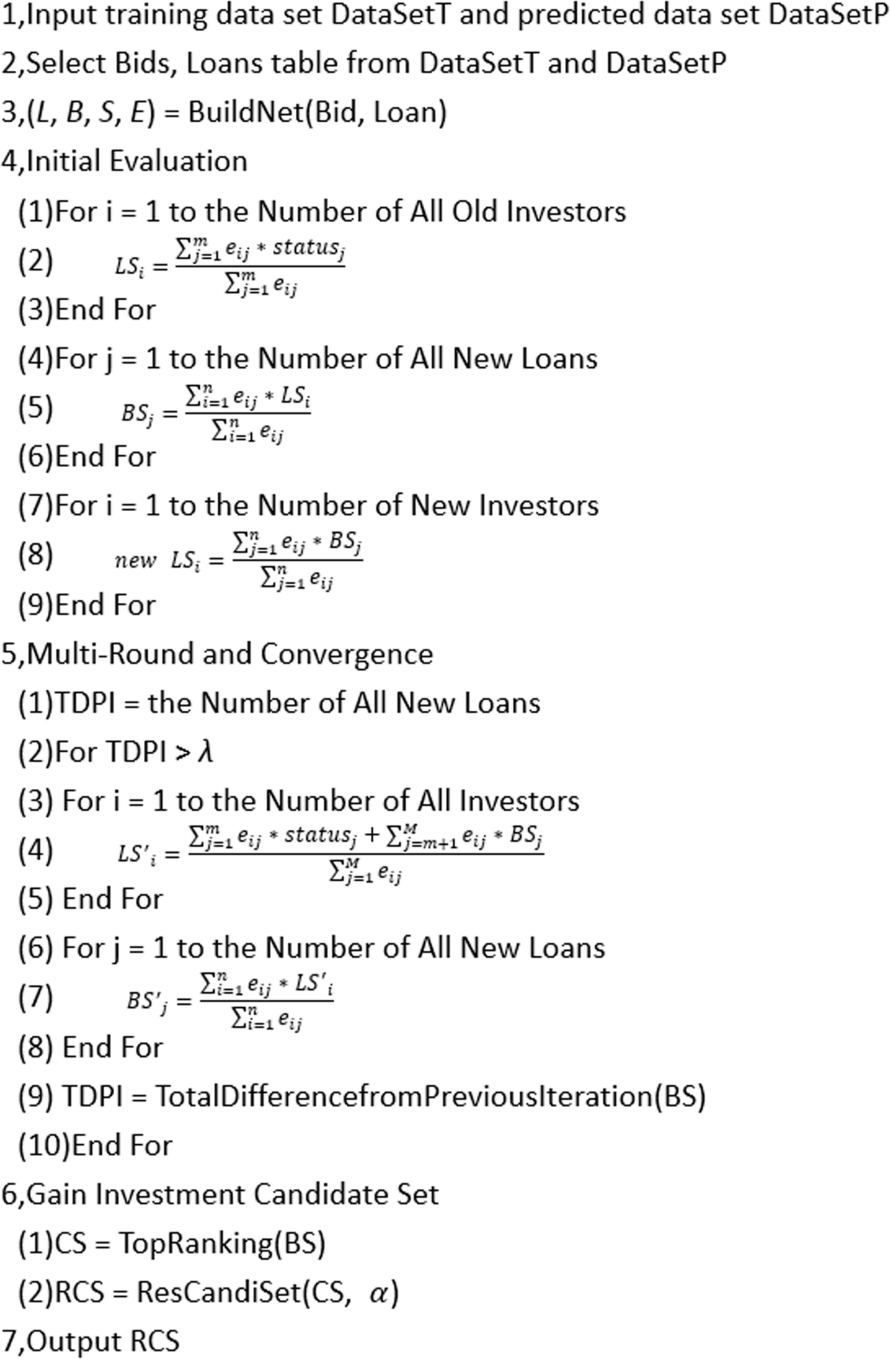
Investment decision process.

Fig 7 describes the detailed process of computation and decision. This process can be divided into four parts. First, in the process from line 1 to line 3, we need to input the training data *DataSetT* and the predicted data set *DataSetP*, which contain the most important information (such as bids and loans). The bid table records each investor's bidding to a loan, one line for each loan. The loan table stores recorded unique loans We use the two types of table to build the *bipartite graph* which depicts the correlation of investors and loans. We also employ these tables to obtain the computation data of *L*, *B*, *E*, *S*, which correspond to the set of investors, borrowers, bidding, and loan status, respectively. Second, initial evaluation begins at line 4. We use Eq (1) to compute the confidence level of all old investors in line 1 to line 3 of the initial evaluation, Eq (2) to predict the paid probability of all new loans in line 4 to line 6, and Eq (3) to estimate the confidence level of new investors in line 7 to line 9. Third, in line 5, the goal of multi-round iteration is to evaluate the underlying confidence level of investors and the paid probability of loans until convergence. We will iterate this process until Total Difference from Previous Iteration (TDPI) converge to *λ* which is set at a reasonable tolerance value. In line 3 to line 5 of Multi-Round and Convergence, we utilize Eq (4) to calculate the confidence level of all investors and employ Eq (5) to estimate the paid probability of all new loans in line 6 to line 8. Finally, we sort the paid probability of all current or bidding loans and generate the result candidate set RCS that are the top α ranking based on the paid probability.

## Experimental results

In this section, we validate our model by employing real-world data on P2P lend platform. We first describe the data that are used in the experiments. In the following sections, we illustrate the effectiveness of the results predicted by the proposed model. We find that our decision model can effectively locate the paid loans.

### Experimental data description

In our experiment, we download the data from Prosper, and select a training data set from January 2009 to June 2010 and a predicted data set from January 2010 to June 2010. We have selected Prosper because it is deemed relatively stable in this period. We seek two types of the most important tables, namely bids and loans to train and test our model.

The bids table contains MemberKey, ListingKey, Amount, and CreationDate. These fields represent unique investor, unique loan, amount of one investor bidding for a loan, and the bid creation date, respectively. A bid is created when a lender wishes to lend money to a borrower. The loans table includes ListingKey, Status, and CreationDate. These fields stand for unique loan, loan status (i.e., current, paid or unpaid), and creation time of the loan, respectively. These information and correlation are useful in building the bipartite network of the relationship of investors and loans.

In our selection data set, the bids table recorded 300,384 recorders and loans table saves 4,335 loans.

### Result analysis

**Fig 8:**
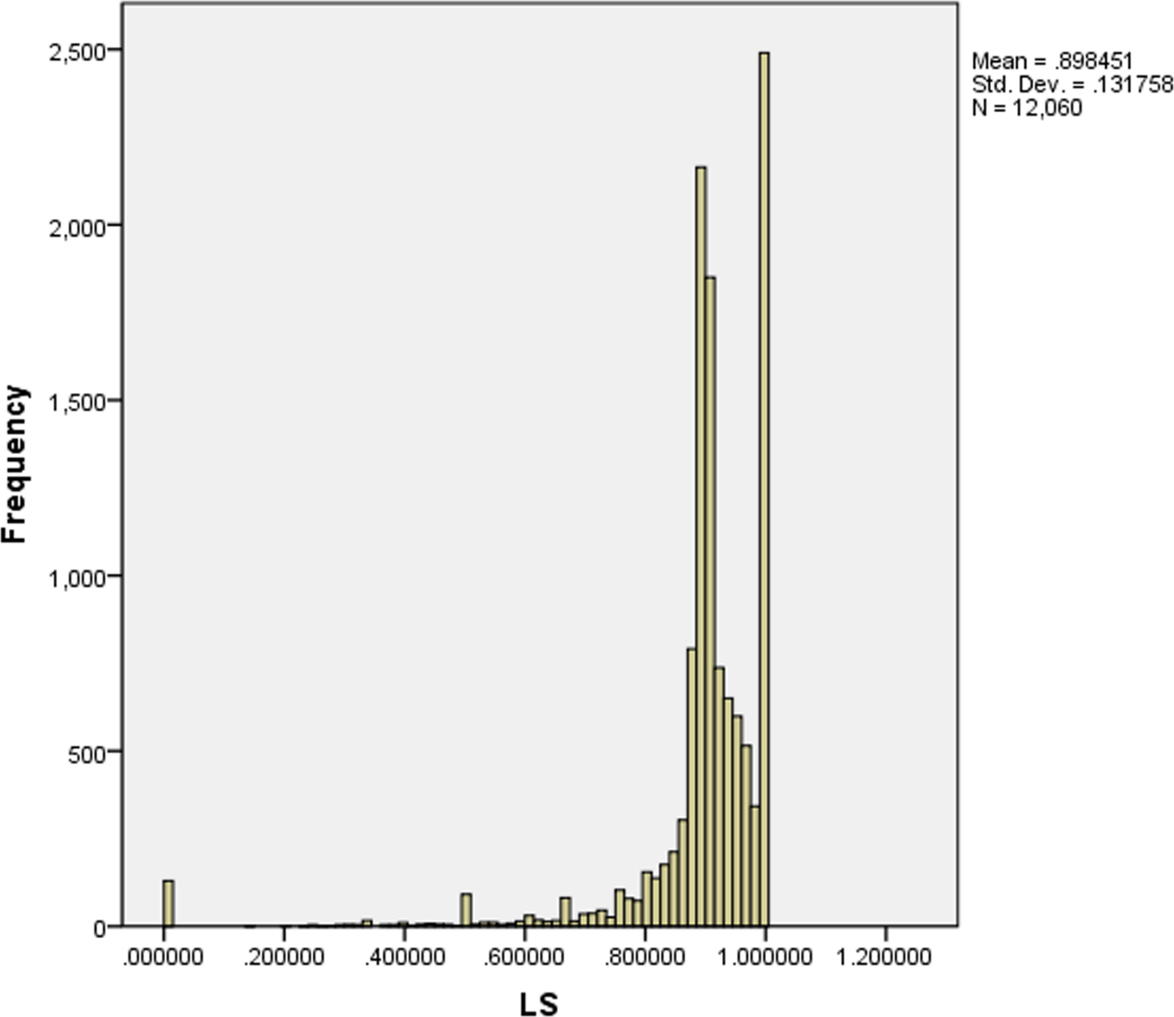
Confidence level of investors.

**Fig 9:**
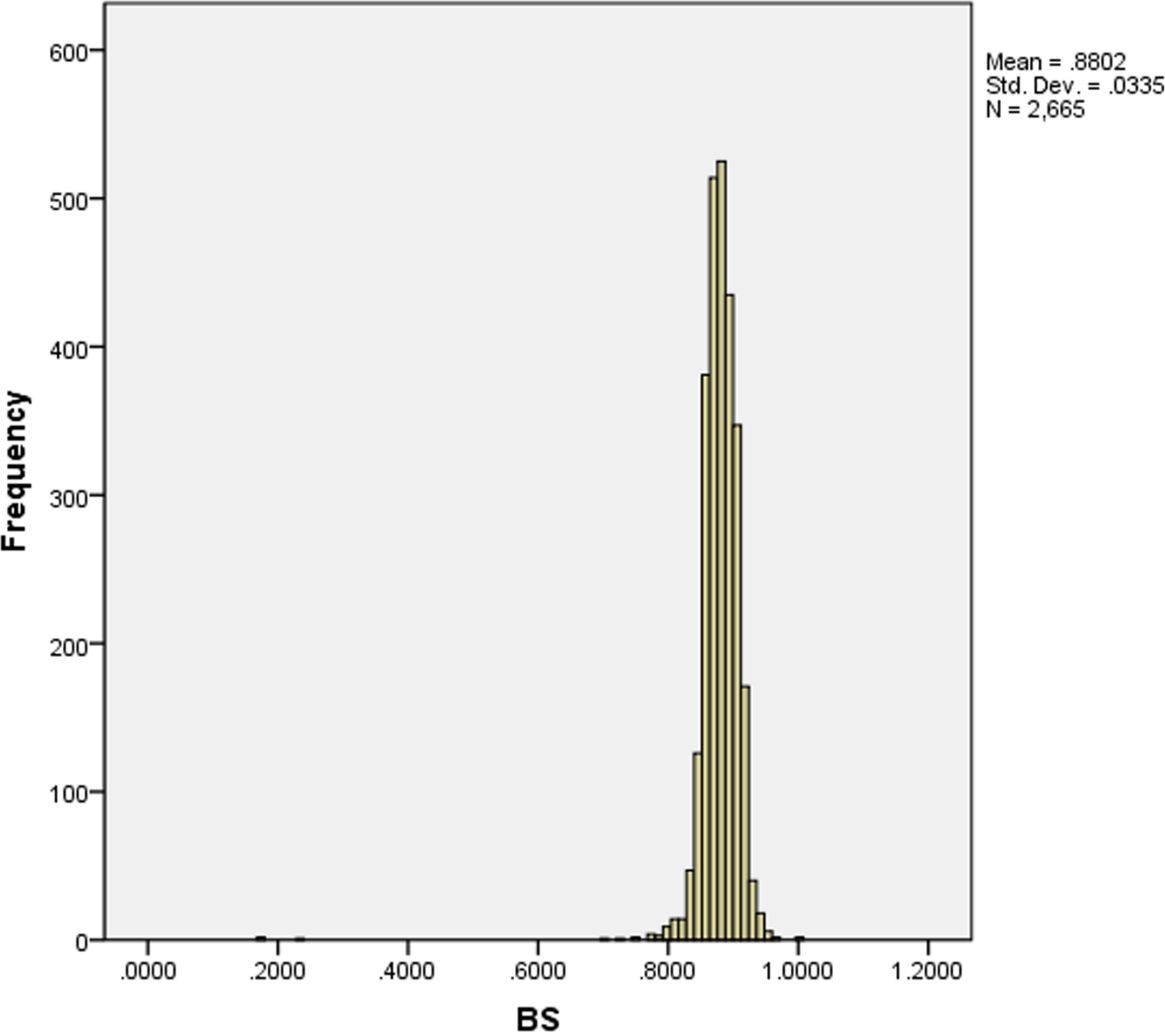
Paid probability of loans.

In the following section, we illustrate how our decision model is validated to predict whether a loan will be paid or unpaid by analyzing computation results. Fig 8 shows the confidence level of the investors’ statistical histogram, whose results converge after 24 iterations. This chart suggests that the confidence level of the majority of investors is less than 0.9 and that a considerable amount of investors have good performance. We employ the confidence level of investors *LS* to predict the paid probability. Fig 9 shows the statistical histogram of the paid probability of loans.

Fig 9 shows that the paid probability of loans presents a Gaussian distribution. Additionally, we utilize Eq (6) to calculate the accuracy rate *AR* at given interval *θ*. Results are given in Table 1.

**Table 1:** Accuracy rate of paid probability interval.

θ	<0.8	0.8to0.82	0.82to0.84	0.84to0.86	0.86to0.88	0.88to0.9	0.9to0.92	0.92to0.94	>0.94
Pain	5	13	63	322	484	456	328	51	14
Total	10	16	75	389	557	508	344	52	15
*AR*	0.5	0.81	0.84	0.83	0.87	0.90	0.95	0.98	0.93

$$AR=\frac{N_{1}}{N_{1}+N_{0}}$$ (6)

Here, *N*_1_ is the number of paid loan, and *N*_0_ is the number of unpaid loan.

**Fig 10:**
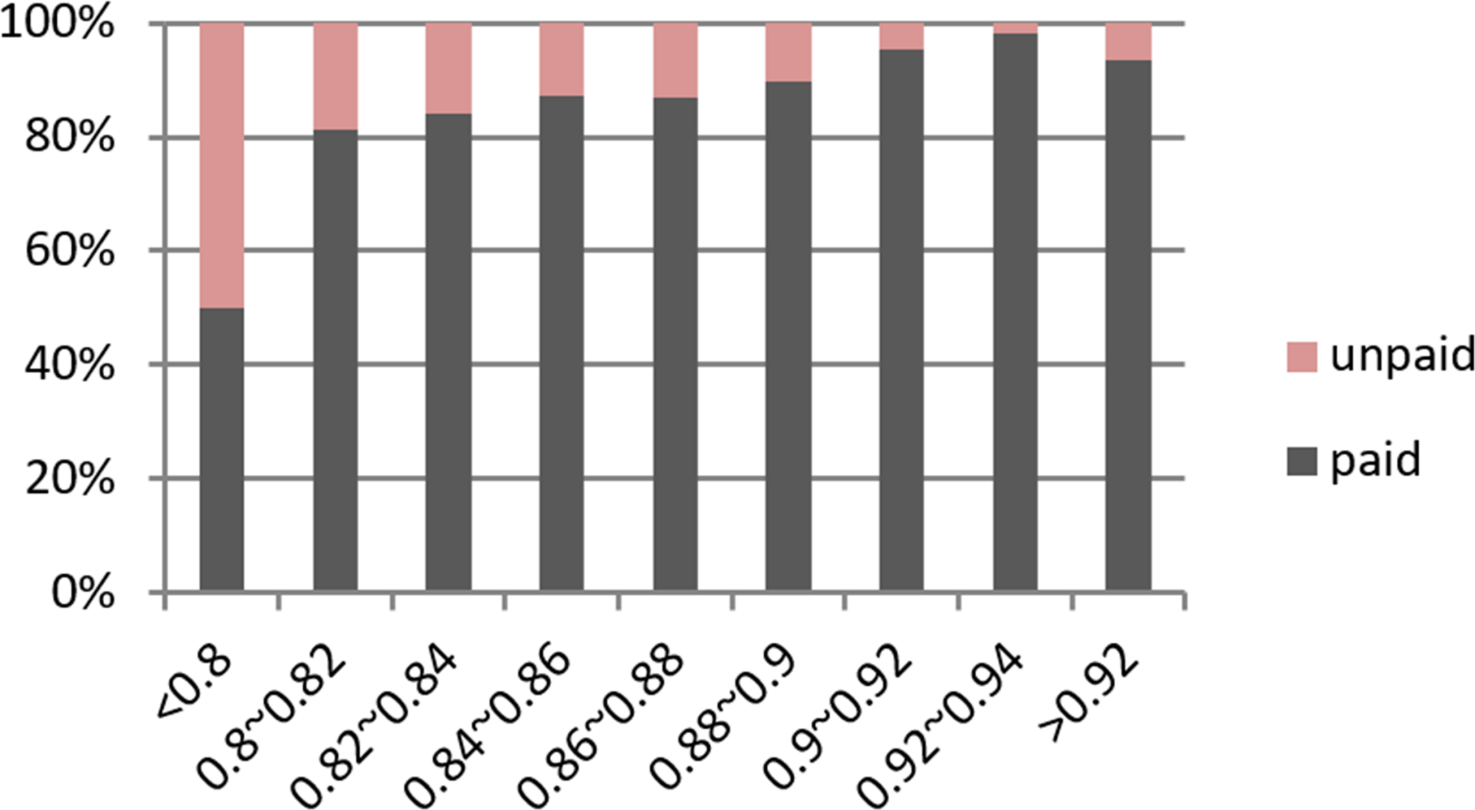
Contrast ratio of paid and unpaid probability of investors.

Table 1 shows that the accuracy rate increases when the degree of paid probability interval increases. Fig 10 illustrates that the unpaid number significantly decreases when the degree of paid probability increases. Therefore, our decision model is effective to help investors select loans. The results also indicates that some potential benefits may be lost if we assign *θ* a very large value.

**Fig 11:**
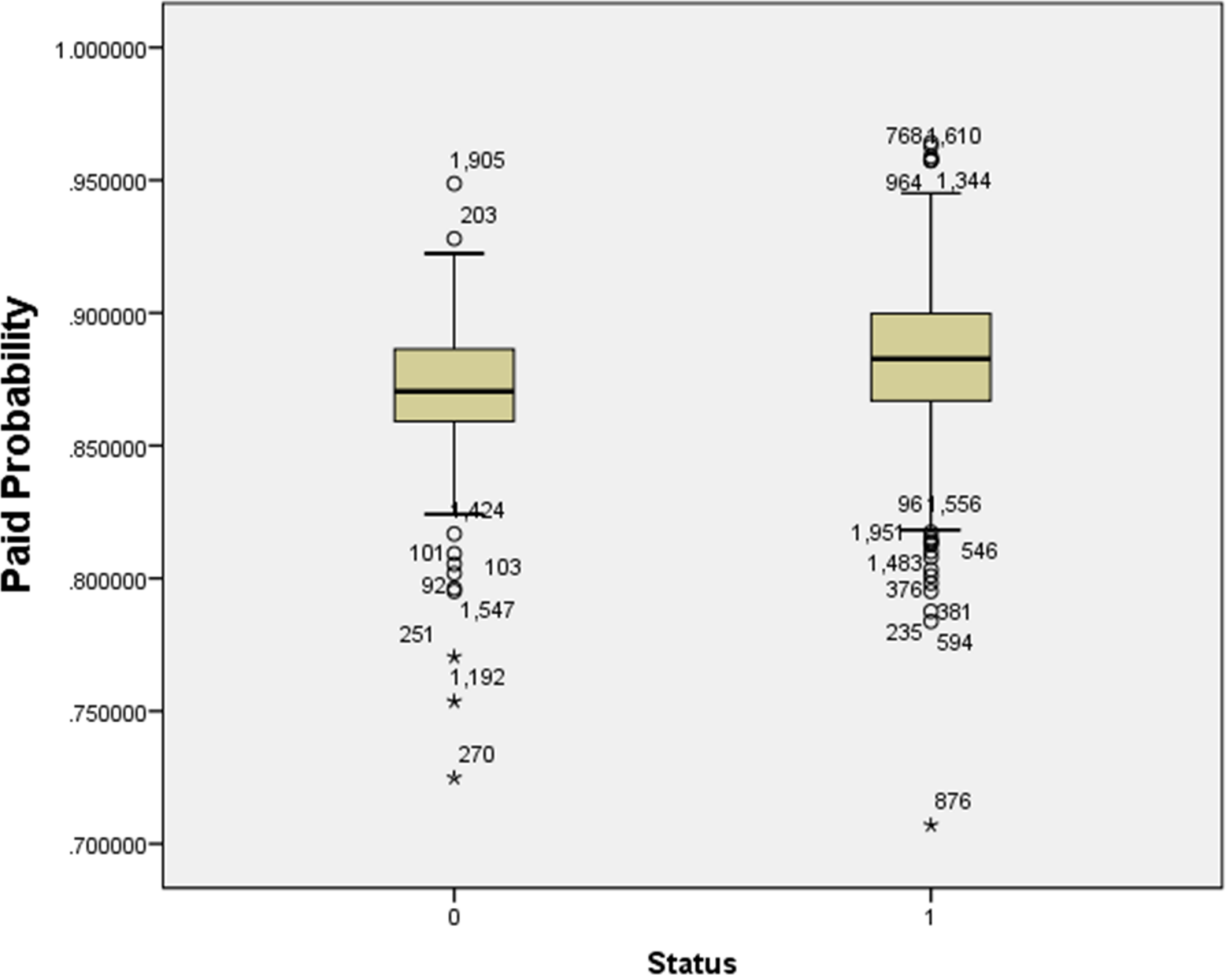
Box plot for paid and unpaid probability investors.

Fig 11 shows that 50% of paid probability is higher than the unpaid. This result is a good sign that validates our decision model.

### Result comparisons

**Fig 12:**
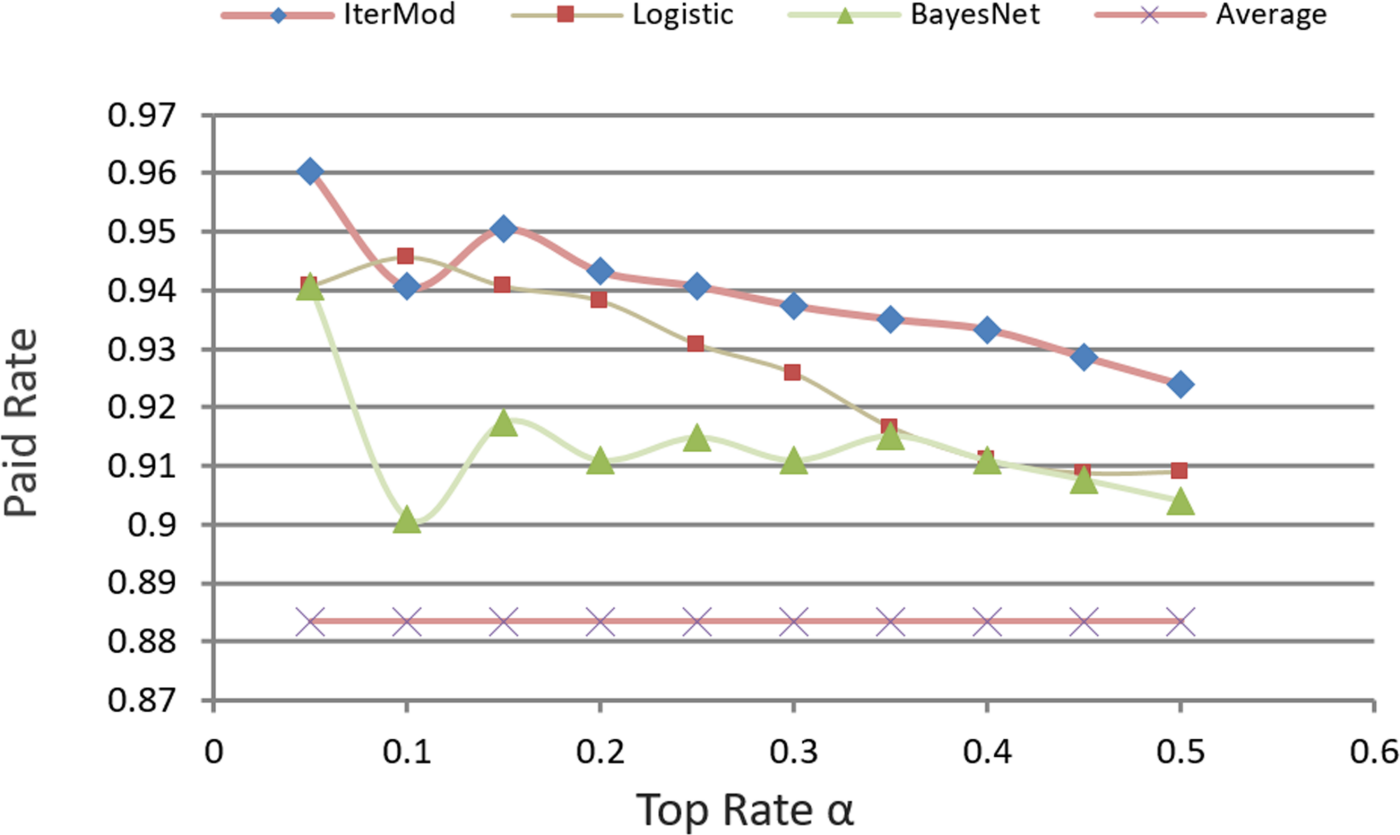
Comparison of paid rate.

In P2P lending, our decision model identifies the ranking of loans, from the best loans to the worse loans, according to the paid probability of loan. Investors can then set a customized definition variable *α* to select the top set as the candidate investment set. Fig 12, compares the paid rate. This figure uses the average paid rate as the baseline. The chart shows that investors make investment decisions in a random way. Approximately 88.3% of loans can be paid, which shows that Prosper is doing a good job in monitoring the transaction process. We compare our candidate selection with logistic regression and BayesNet, which are the most commonly used methods on personal loans risk analysis. For Logistic Regression and BayesNet, loans are ranked in the decreasing order of the expected paid rate. We can explicitly see that the evaluation result of our decision model is more effective than that of Logistic Regression, BayesNet, and Average. Although a slight decline relative to Logistic Regression exists when α = 0.1, the candidate set chosen by our model still has higher paid rate than that of others.

### IterMod and logistic hybrid

Fig 12 shows that BayesNet and IterMod are singular when α equals 0.1. On the other hand, Logistic is more active than the other two. We hypothesize that Logistic will achieve better performance if mixed with the computation model of IterMod. To integrate the two classification models, we introduce Eq (7). Thus, the paid probability BS_i_ of loan b_i_ consists of its confidence level from IterMod and Logistic.

$${BS}_{i}=\theta {IBS}_{i}+(1-\theta ){LBS}_{i}$$ (7)

Here, *θ* is the confidence weight that represents the confidence level for the result of IterMod.

**Fig 13:**
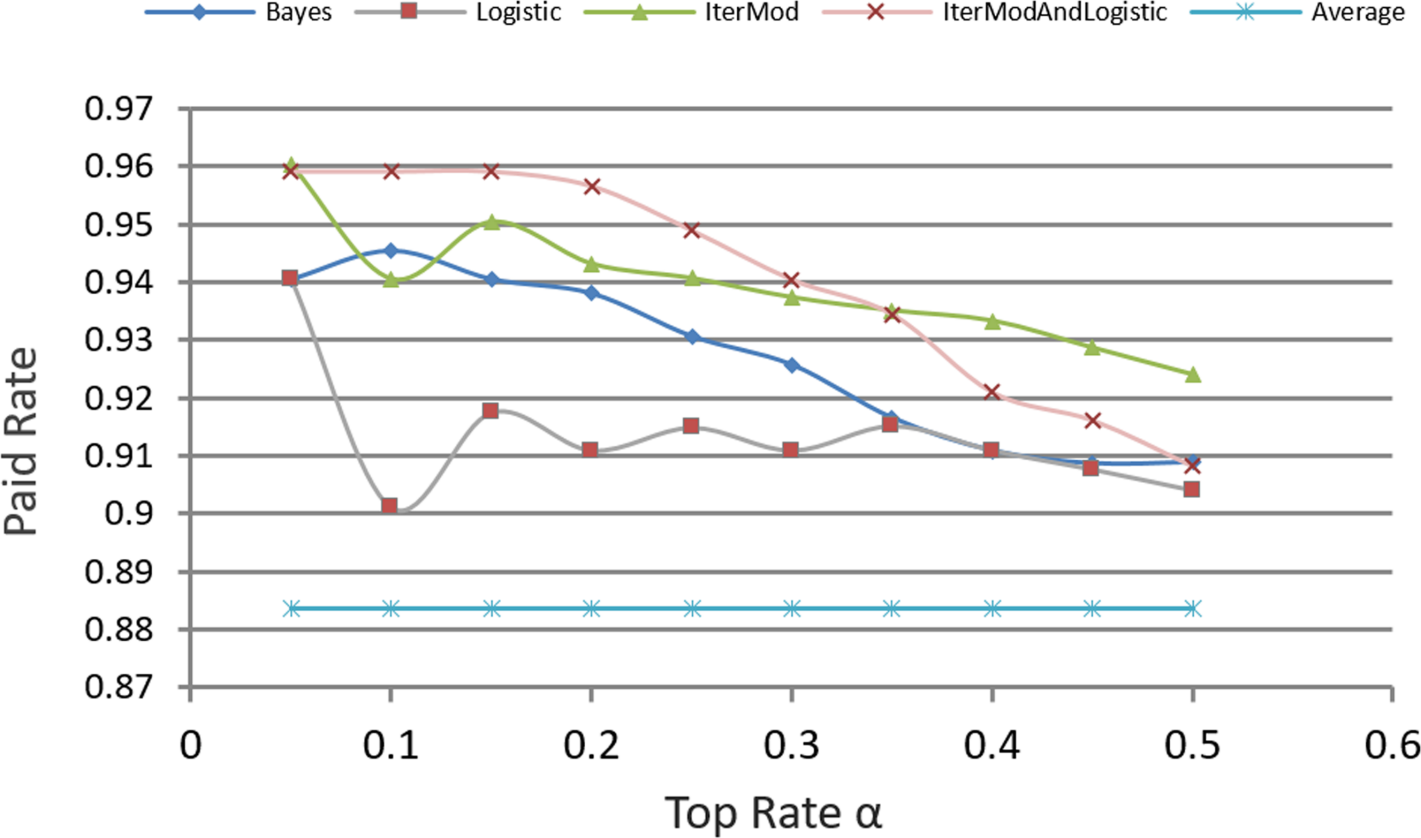
Comparison of paid rate of models.

Fig 13, compares the paid rate of the hybrid model of IterMod and Logistic with other models. Results show that the hybrid model of IterMod and Logistic is efficient and has a stable performance for making investment decisions. In this experiment, we set *θ* as 0.6. This is because experimental results show that iterative computation model achieves better performance than Logistic. Based on this comparison result, we conclude that Logistic and our computation model are complementary to each other.

**Fig 14:**
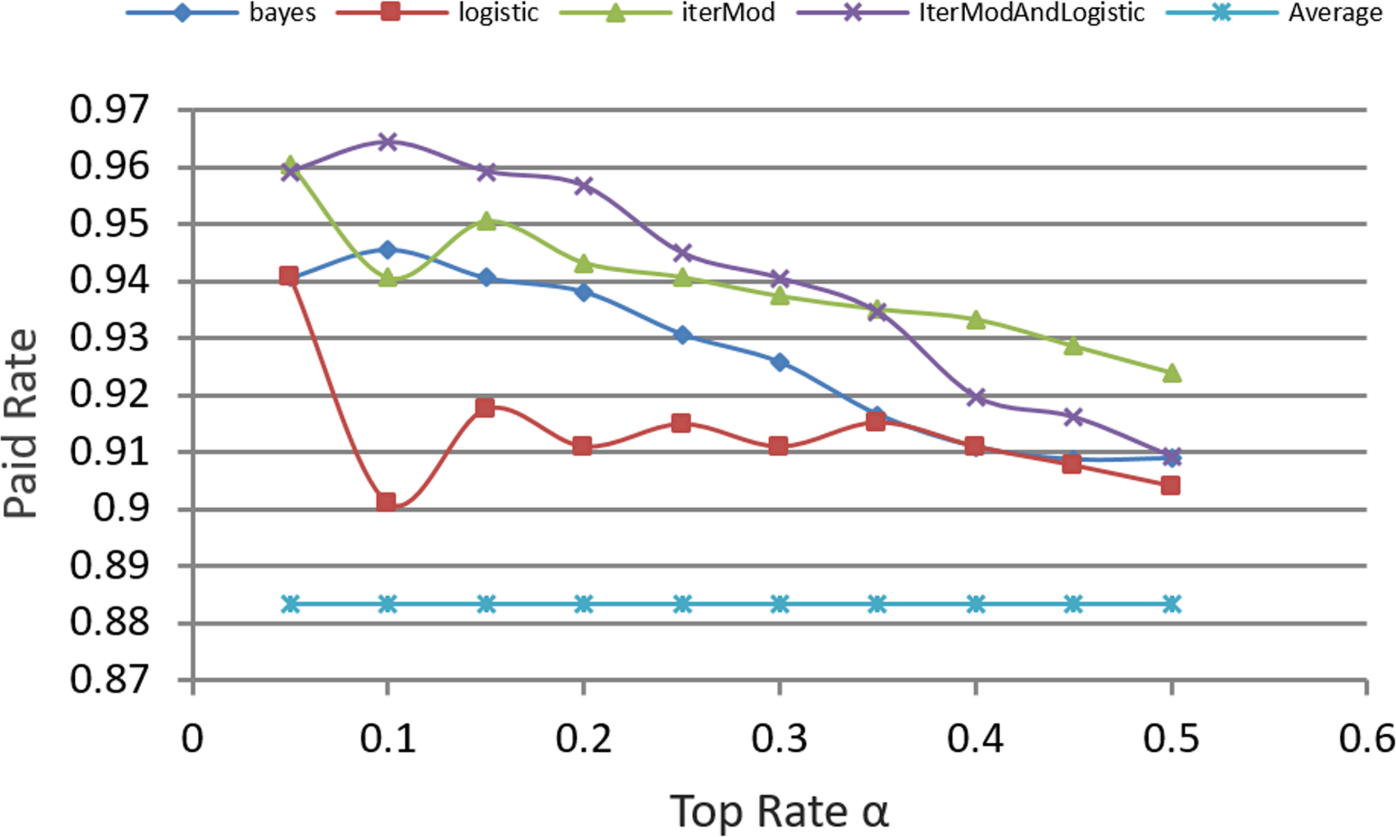
Comparison of paid rate of *θ* = 0.5.

Fig 14 shows the comparison result when we set θ = 0.5. The figure also verifies our hypothesis that the hybrid classification model can obtain better performance compared with other models. The most remarkable point is α = 0.1, where IterMod's, BayesNet's, and Logistic's performance are singular, Logistic acts as an important complement in the hybrid model to improve the investment decision. Therefore, our computation model and Logistic complement each other, and the hybrid model by integrating both approaches is more efficient than each of the individual model alone.

## Concluding remarks

In this paper, we propose an investment decision model on P2P lending. We exploit the investment performance of old investors on the basis of their confidence level, predict new investors' confidence level, and estimate current or bidding loans' paid probability. Specifically, we employ historical records to compute the confidence level of old investors. Using the confidence level of old investors, we estimate the paid probability of new loans, which in return is utilized to predict the confidence level of new investors. Subsequently, we utilize the paid probability and the status of all historical and new loans to calculate the confidence level of all investors. This confidence level is then used to estimate the paid probability of all new loans. Finally, we iterate the process until the confidence level of all investors and all new loans converges. Experimental results on real-world P2P lending data demonstrate that our decision model can effectively filter out reliable borrowers and significantly improve the investment performance of investors. Experiments also show that the Logistic classification model and our iteration computation model complement each other, which inspired us to integrate the two classification models. Experiments show that the hybrid model (i.e., the integration of iterative computation model and Logistic classification model) is more efficient and stable than the individual model alone. In the future, an interesting work is to investigate P2P lending by considering multi-objective evolutionary algorithms (MOEAs) [23], since the effectiveness of MOEAs have been verified on a variety of real-world problems [24–26]. And machine learning classification model [27–29] such as the state-of-art v-support vector machine methods [30–32], dimension reduction techniques [33–35], neural-like computing models [36–38] and spiking neural networks [39, 40] could be tested. Community detection methods, especially overlapping communities detection [41] also can be considered for the further improvement.
